# Radiographic bone level around particular laser-treated dental implants: 1 to 6 years multicenter retrospective study

**DOI:** 10.1186/s40729-020-00230-w

**Published:** 2020-07-28

**Authors:** C. Mongardini, B. Zeza, P. Pelagalli, R. Blasone, M. Scilla, M. Berardini

**Affiliations:** 1 Department of Maxillo-Facial and Odontostomatologic Sciences, University “La Sapienza” of Rome, Rome, Italy; 2 Department of Dentistry, Section of Periodontology, Albanian University, Tirana, Albania; 3 Roma, Italy; 4 Udine, Italy; 5 Arezzo, Italy; 6 Pescara, Italy

**Keywords:** Dental implants, Osseointegration, Laser surface, Implant survival rate

## Abstract

**Purpose:**

The aim of the present retrospective study was to evaluate clinical and radiological outcomes, in terms of implant survival rate, marginal bone loss, and peri-implantitis incidence, of a titanium implants with an innovative laser-treated surface.

**Materials and methods:**

A total of 502 dental implants were inserted in four dental practices (Udine, Arezzo, Frascati, Roma) between 2008 and 2013. All inserted implants had laser-modified surface characterized by a series of 20-μm-diameter holes (7–10 μm deep) every 10 μm (Synthegra®, Geass srl, Italy). The minimum follow-up period was set at 1 year after the final restoration. Radiographs were taken after implant insertion (T0), at time of loading (T1), and during the follow-up period (last recall, T2). Marginal bone loss and peri-implant disease incidence were recorded.

**Results:**

A total of 502 implants with a maximum follow-up period of 6 years were monitored. The mean differential between T0 and T2 was 0.05 ± 1.08 mm at the mesial aspect and 0.08 ± 1.11 mm at the distal with a mean follow-up period of 35.76 ± 18.05 months. After being in function for 1 to 6 years, implants reported varying behavior: 8.8% of sites did not show any radiographic changes and 38.5% of sites showed bone resorption. The bone appeared to have been growing coronally in 50.7% of the sites measured.

**Conclusion:**

Implants showed a maintenance of marginal bone levels over time, and in many cases, it seems that laser-modified implant surface could promote a bone growth. The low peri-implant disease incidence recorded could be attributed to the laser titanium surface features that seem to prevent bacterial colonization. Future randomized and controlled studies are needed to confirm the results of the present multi-centrical retrospective analysis.

## Introduction

Dental implant-supported rehabilitations are safe and predictable therapies [1–5] whose rising demand varies according to the patient population ages [6]. Clinicians are in continuous search for solutions to minimize biological and mechanical complications related to the implants over the time.

Patients’ susceptibility to periodontitis, cigarettes smoke [7], and the implant surface features [8] seemed to be the parameters closely related to the early onset of peri-implant diseases after implant osseointegration. It was reported [9] that a variable percentage, ranging from 8.6 to 14.4%, of restored implants are easily affected by peri-implantitis within 5 years after functional loading. A more recent literature review [10] revealed an implant survival rate of 97.3% after 5 years or more of loading with less than 5% of the implants affected by peri-implantitis.

Achievement of implant stability and maintenance of stable crestal bone level are prerequisites for a successful long-term function of oral implants [11]. In non-pathological conditions, after implant insertion and prosthetic loading, marginal bone loss appears more pronounced during the first year in function continuing slowly thereafter. It has been assumed that marginal bone resorption around implants represents a reaction to treatment and is not at all a disease process rather than an initial foreign body response to the implant [12]. The amount of initial crestal remodeling has been observed to be related to host factors, implant design, surgical protocol, and restorative protocols [13]. The prevalence and reasons for crestal bone loss during functional load are well documented [14] in the literature, but it is still unclear, owing to the great number of factors. Implant geometry, surface features [10], neck design [15], or the micro-gap between fixture and abutment seemed to be all involved in peri-implant bone remodeling phenomena. A recent review demonstrated that crestal bone levels are better maintained in the short-medium term when internal fixture-abutment connections are adopted, and among them, conical connections seem to be more advantageous, showing lower peri-implant bone loss [16].

The long-term implant success rate could also be influenced by other factors such as patient systemic disease [17], tobacco smoke [18], untreated periodontitis [19], surgical technique [20], host bone density [21], fixture macro- and micro-geometry [22], and implant surface [23–25].

It seems that surface topographies are a modifiable factor that influence physiologic and pathologic marginal bone loss, and different implant surfaces have been developed over the years in order to increase the speed of bone apposition during osseointegration phase and prevent bacterial adhesion [26].

An innovative laser-modified implant surface seems to be able to promote titanium osteointegration and, at the same time, to inhibit biofilm formation compared to sandblast surface. The laser used to create this surface was pulsed by a diode-pumped solid state (DPSS) source laser, in a Q-Switch output rate. The DPSS Nd:Yag Q-sw laser is characterized by a very high speed and flexibility in this type of work. In fact, it is possible to carry out precise and repeatable micro-workmanship with micrometric tolerance, allowing the application of technology even in very inclined areas of the surface. With this technique, the material is removed from the surface as vapor and this “cold” ablation assures a “clean” finish without thermally altered areas, without the formation of cracks, and with good repeatability of the process [27].

Many authors have previously investigated this innovative titanium surface treatment using in vitro and in vivo animal studies, but before the present paper, no study published human clinical outcomes.

An in vitro study [28] evaluated and compared the amount of biofilm produced by *Staphylococcus aureus*, *Pseudomonas aeruginosa*, and *Porphyromonas gingivalis* on conventional sandblasted titanium and on laser-treated surface. Results showed a lower biofilm production on laser-modified surface compared to the sandblasted one. Other authors [29] demonstrated a higher albumin and fibronectin adsorption compared to sandblast or machined surfaces, and it is reported to have an average bone loss of 0.73–0.84 mm the first year in function.

Despite the promising properties, little evidence is available on longer-term function of this type of implants.

The aim of the study was to evaluate the marginal bone level, implant survival rate, and peri-implant prevalence of this particularly laser-modified implant surface.

## Materials and methods

This retrospective study was conducted by analyzing X-rays from patients treated with at least one way Milano implants with Synthegra® laser-treated surface (Geass srl, Pozzuolo Del Friuli, Udine, Italy) from four private practices in Italy (Udine, Arezzo, Frascati, Roma) between 2008 and 2013. The protocol followed the principles of the Declaration of Helsinki and was approved by the Ethics Committee of Sapienza University (Rome, Italy) (ref. 3339/27.11.2014). All patients signed the informed consent on the use of personal information related to the aim of the study.

**Fig. 1:**
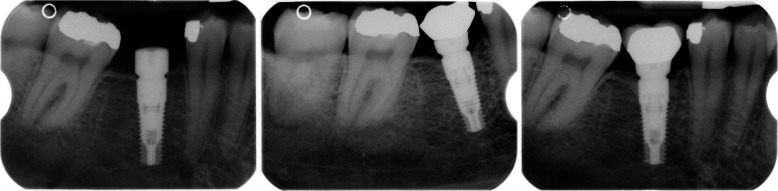
Illustrative case of radiological follow-up period in the mandible. T0 in the left image, T1 in the central, and T2 in the right

**Fig. 2:**
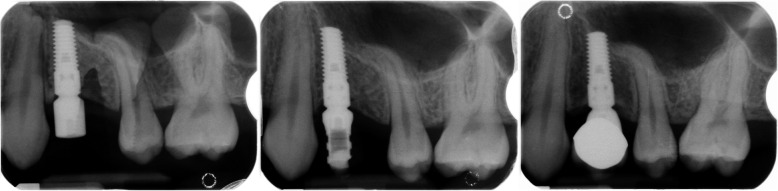
Illustrative case of radiological follow-up period in the upper jaw. T0 in the left image, T1 in the central, and T2 in the right

**Fig. 3:**
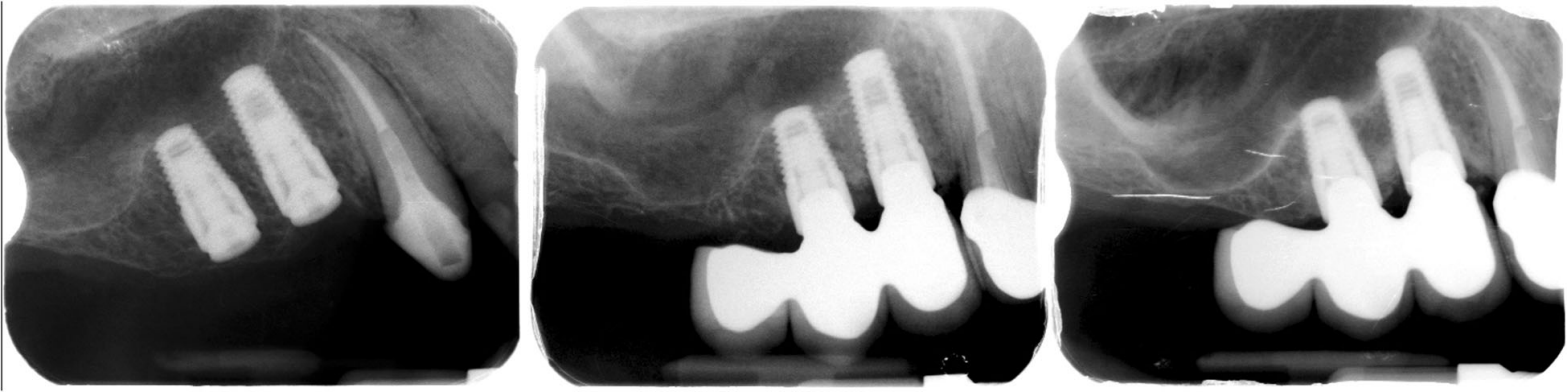
Illustrative case of radiological follow-up period of two adjacent implants in the maxilla. T0 in the left image, T1 in the central, and T2 in the right

No restriction on systemic or local characteristics was applied other than those necessary for undergoing an oral surgery procedure of dental implant insertion by an expert in implantology. The only inclusion criteria were at least one Synthegra® dental implant and clear peri-apical X-rays of implant insertion (T0), time of loading (T1), and last recall (T2). The time of loading (T1) coincided with the final restoration delivery. Some exemplificative cases are shown in Figs. 1, 2, and 3. The last recall was restricted to a minimum of 1 year after restoration delivery. Implants with incomplete radiological documentation were excluded from the study.

All patients were treated with the same dental implant type following the manufacturer’s drill sequence for the osteotomies using sharp instruments; drill speed was between 700 and 1000 rpm under abundant sterile saline irrigation to minimize bone trauma.

The implant diameters used, compared to the total, were 2.14% for 3.4 mm, 38.33% for 3.8 mm, 53.57% for 4.5 mm, and 5.95% for 5.5 mm. The most used fixture length was the 12 mm (23.10% of cases) followed by the 11 mm (19.29% of cases), 13 mm (18.33% of cases), 15 mm (15.71% of cases), 10 mm (13.33% of cases), 9 mm (8.33% of cases), and 8 mm (1.90% of cases).

The following parameters were monitored during the follow-up period and utilized to establish the peri-implantitis diagnosis: bleeding on probing presence, suppuration presence, probing pocket depth beyond the fixture-abutment connection level, and crestal bone loss more than 2 mm in respect to the bone level at baseline (final restoration deliver).

The maintenance implant protocol included recall visit every 6 months with professional oral hygiene and measurement of clinical parameters such as plaque index (PI), full-mouth plaque score (FMPS), and full-mouth bleeding score (FMBS).

The health scale specific for endosteal implants, according to the ICOI Pisa Consensus Conference [30], was used in the present study in order to classify implant into categories of success, survival, or failure. The implant success has been associated to pain absence, 0 mobility, < 2 mm radiographic bone loss from initial surgery, and no exudates history. Implant satisfactory survival has been associated to pain absence, 2 to 4 mm of radiographic bone loss, and no exudates history.

The method used for bone measurements was described in detail in Zeza et al. [31]. Briefly, mesial and distal marginal bone level was recorded for each implant. Conventional periapical radiographs were obtained using the long-cone paralleling technique when the implant was put in function and on the most recent visit. Acceptable radiographs (visible full length of the implant and clearly distinguishable implant threads) were imported to the software used for bone-level measurements (CSN Image Database®, Version 3.14, ArchiMed, Turin, Italy). Following image and measurement calibration, based on actual implant length as recorded in the chart, the original image size was enlarged × 1.5 and the implant platform shoulder was used as reference point for bone level measurements. Mesial and distal bone level measurements, performed by a trained and calibrated examiner (BZ), were recorded and analyzed separately. Examiner reliability was assessed by performing duplicate measurements, 1 week apart, on 40 randomly chosen distinct radiographs; difference between duplicate measurements was < 0.5 mm. Radiographic distance between bone crest and implant platform more than 2 mm were identified as peri-implantitis bone loss.

Bone level measurements were organized in Excel and transferred to Stata13.1 (StataCorp LP; College Station, TX, USA). Descriptive analysis was performed to summarize the general information on patient level and determine the bone level around implants at the two different time periods.

## Results

All implants had a small thread design, with smooth neck of 0.25 mm in the most coronal area and a micro-thread collar of 3.25 mm in length. The thread angle was 60°, and the screw pitch was 0.6 mm. The fixtures showed a 22° conical internal hexed prosthetic connection with platform switching. The conical portion height was 1.5 mm for 3.4/3.8 implant diameters and 2.2 mm for 4.5/5.5 implant diameters while the hexagon height was 1.4 mm for all implants.

All implants showed a laser-treated surface characterized by a series of 20-μm-diameter holes (7–10 μm deep) every 10 μm. The *R*_a_ value was 0.37 μm (value obtained considering the holes not as part of the roughness but as part of the primary profile. *R*_a_ inside the holes is 0.1 μm while outside the holes is 0.4 μm). Implants had internal hexagon associated to a conical connection.

A total of 502 implants, inserted in 263 patients, were monitored with a maximum follow-up period of 6 years (Table 1). Patients’ mean age was 60 ± 12 years. The distribution of implant sites is summarized in Table 2.

**Table 1:** Number of patients treated and implants inserted in each center of the present retrospective multi-centric analysis

	Center 1	Center 2	Center 3	Center 4	Total
Patients no.	75	65	79	44	263
Implants no.	173	101	146	82	502

**Table 2:** Distribution of implant sites

Implant sites	Center 1	Center 2	Center 3	Center 4	Total	%
**Upper molars**	19	13	21	23	76	15.20
**Upper premolars**	50	41	49	28	168	33.60
**Upper canines**	1	8	9	1	19	3.80
**Upper incisives**	15	13	14	5	47	9.40
**Lower molars**	53	17	26	8	104	20.80
**Lower premolars**	30	9	17	9	65	13.00
**Lower canines**	1	0	5	2	8	1.60
**Lower incisives**	5	0	5	3	13	2.60
					500	100.00

Nine implants failed the osseointegration after 3 months, and the mean implant survival rate was about 98.1% while the mean implant success rate was 91.5%. Among failed implants, seven implants were inserted in the upper molar region.

After being in function for 1 to 6 years, implants reported varying behavior. While 8.8% of sites did not show any radiographic changes, 38.5% of sites showed bone resorption. The bone appeared to have been growing coronally in 50.7% of the sites measured. In center 4, greater bone loss than other centers was detected; this is probably caused by dependent operator variables.

Overall, only 8% of mesial sites and 10% of distal sites showed a bone resorption more than 2 mm. The prevalence of peri-implantitis was 5.8% at site level and 6.5% on implant level because only some sites with bone loss > 2 mm showed also bleeding on probing and suppuration.

**Fig. 4:**
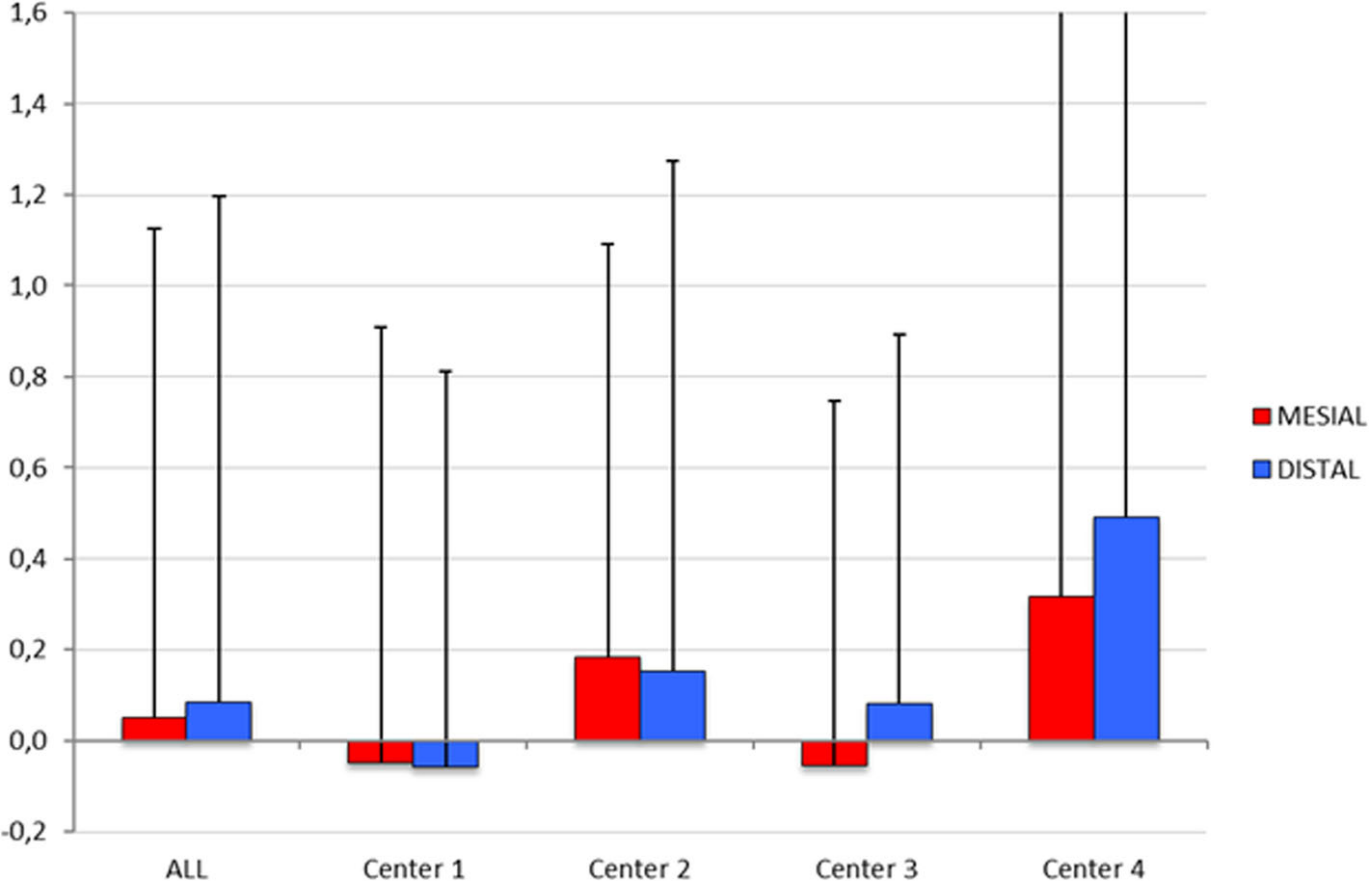
Data distribution of marginal bone loss/gain between T0 (implant insertion) and T2 (last recall) of each center

The mean differential of marginal bone loss between T0 (implant insertion) and T2 (last recall visit) was 0.05 ± 1.08 mm at the mesial aspect and 0.08 ± 1.11 mm at the distal with a mean follow-up period of 35.76 ± 18.05 months (Tables 3 and 4). The data distribution of each center is illustrated in Fig. 4.

**Table 3:** Mean differential of peri-implant bone loss/gain (mesial and distal) between T0 (implant placement) and T2 (last recall). The minimum follow-up period was set at 1 year post-functional load

	∆Mesial mm	Dev st	∆Distal mm	Stan dev	Mean loading time (months)	Stan dev
Center 1	− 0.05	0.96	− 0.06	0.87	34.25	14.97
Center 2	0.18	0.91	0.15	1.12	33.03	19.31
Center 3	− 0.06	0.80	0.08	0.81	43.28	18.94
Center 4	0.32	1.61	0.49	1.80	35.97	19.84
**Total**	**0.05**	**1.08**	**0.08**	**1.11**	**35.76**	**18.05**

**Table 4:** Mean peri-implant bone loss/gain between T1 (time of loading) and T2 (last recall) in each year post-functional load

Loading Time	∆Mesial mm	Stan dev	∆Distal mm	Stan dev	Mean loading time (months)	Stan dev
**> 2 years**	0.05	1.11	0.13	1.18	44.31	14.44
**> 3 years**	0.08	1.20	0.14	1.27	51,23	11.41
**> 4 years**	0.13	1.16	0.23	1.27	58.18	8.45
**> 5 years**	0.24	1.23	0.39	1.34	64.92	6.13
**> 6 years**	0.16	0.69	0.11	0.58	74.25	2.43

## Discussion

It was well documented [32, 33] that titanium surface composition, hydrophilicity, and roughness are parameters which may play a key role in implant–tissue interaction and osseous integration.

Data from the present investigation demonstrated that bone level around laser-modified implants in function for 36 ± 18 months showed a mean change of 0.05 ± 1.8 mm for mesial sites and − 0.08 ± 1.11 mm for distal sites. Despite the mean values giving the impression of an overall tendency of a growth of bone around this type of implant surface, only 50.7% of sites behaved that way.

However, the maintenance of marginal bone levels in time was similar and in some cases even better than the values reported in the literature for the most commonly known implants [34, 35]. The values of bone change during the selected period of time showed different tendency and were considerably lower than previously reported studies on the same implant surface. A bone loss of 0.73 mm the first year in function was reported by Felice et al. [36]. A recent retrospective study on 174 patients [37] that evaluated the marginal bone loss around implants with laser micro-grooved collar found mean peri-implant bone resorption of 0.18 ± 0.7 mm at the mesial aspect and 0.19 ± 0.6 mm at the distal aspect. Similar results were also reported from the study of Acharya et al. [38], in which the authors performed an exploratory analysis of annual rates of peri-implant marginal bone loss using the same three radiographical intervals used by the present retrospective study (immediately post-implant placement, time of loading, at least 1 year post-loading), and they found an overall peri-implant marginal bone loss of 0.21 mm/year. Regarding other implant surfaces and systems, the pooled mean marginal bone level change amounted to − 0.24 mm (95% CI − 0.345, − 0.135) for the Astra Tech Dental Implant System, 0.75 mm (95% CI − 0.802, − 0.693) for the Brånemark System, and 0.48 mm (95% CI − 0.598, − 0.360) for the Straumann Dental Implant System over 5 years [39]. Another study evaluating peri-implant bone level changes around surface-modified implants reported a mean bone loss of 0.36 mm from the time of implant placement for implants in function for a mean 32 months [40].

A literature review study, on peri-implant bone loss over the time [41], that examined 758 international studies found an implant survival rate after 12 months of healing of 97.4% for the maxilla and 99.6% in the mandible. The authors reported that the peri-implant crestal bone loss, after 1 year of functional prosthetic load, ranged from 0.43 to 1.13 mm.

**Fig. 5:**
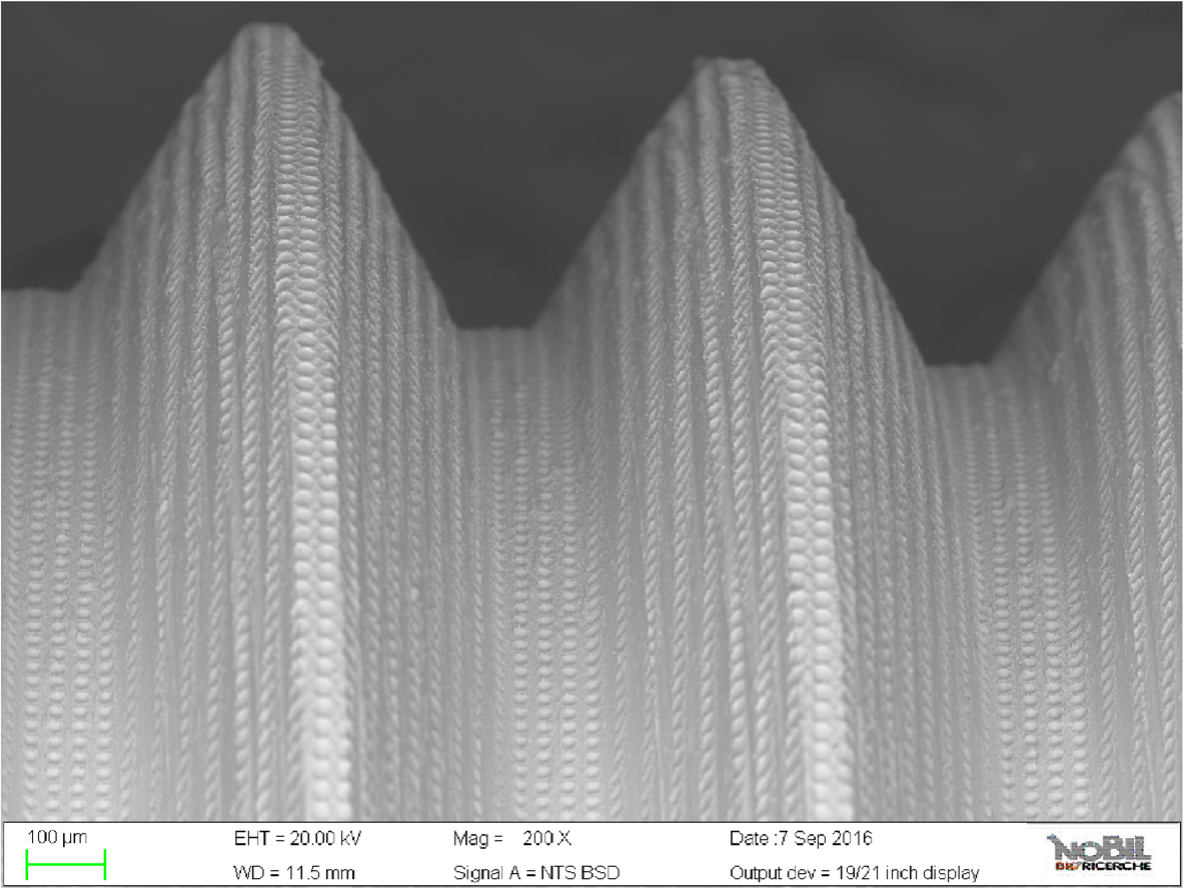
Scanning electron microscopy (SEM) picture of Synthegra® surface. The laser surface is characterized by a series of 20-μm-diameter holes (7–10 μm deep) every 10 μm

The laser technique used allowed to create a pure titanium surface without any contamination (no contact between titanium and the machinery during the production), inorganic residuals from blasting procedure, or residual acid that could pollute the titanium surface purity. The laser surface used is characterized by a series of 20-μm-diameter holes (7–10 μm deep) every 10 μm (Fig. 5).

Sinjari et al. [42] evaluated the effects of different titanium surface treatments on blood clot formation, and they demonstrated in vitro that the laser-conditioned surface, although it has a low roughness value (*R*_a_ of 0.25  ±  0.02 μm) compared to a standard grit-blasted surface (*R*_a_ of 1.30  ± 0.03 μm), had higher wettability and blood clot extension in respect to machined and rough surfaces.

It has been speculated that the initial peri-implant bone reaction could be rather a response to a foreign body by Albretksson et al. This assumption has the support of in vitro results of Quabius et al. [43], observing an enhanced expression of IL-8 when the human blood is in contact with dental implants. Similarly, but in vivo results, Salvi et al. [44] report levels of MMP-8 activity at the implant level higher at all the time points, even in healthy conditions, compared to the tooth level during the development of 21 days experimental mucositis and gingivitis. Both biomarkers are involved in periodontal tissue destruction during inflammation. In addition, the same authors concluded that the peri-implant tissue response is stronger to plaque accumulation than periodontal tissues. Bone loss at the first year in function was reported to be 0.02 mm [45] compared to previously accepted physiological bone loss of 1.5 mm [46]. Cases of regrowth of bone around dental implants following the first year in function, as in the present study, contradict all this theory and indicate the need for further examination and epidemiological evaluation of similar patients.

Trisi et al. [47] demonstrated, in an in vivo animal study, that laser-treated implants had significant higher bone to implant contact percentages (%BIC) and reverse torque values in respect to machined implants. Other authors [48] found no significant differences in %BIC values comparing laser-treated and sandblasted/acid-etched implants in sheep.

High implant success rate from the present study seemed to confirm the results showed by these cited studies.

Peri-implant diseases are infective complications of surrounding dental implant tissues that often occurs some years after the final prosthetic restoration placement. They represent a high risk of implant failure. A recent study, with a long follow-up [49], demonstrated a peri-implantitis incidence of 7.9% on implant level.

Pjetursson et al. [50] studied the 5- and 10-year survival of implant-supported fixed dental prostheses (FDPs) analyzing 32 studies that matched the criteria for the systematic review. They found a survival rate of implants supporting FDPs of 95.6% after 5 years and 93.1% after 10 years, but they observed that, when machined surface implant data were excluded from the analysis, the survival rate increased to 97.2% after 5 years.

The low incidence of peri-implantitis (less than 7%) observed in the present retrospective study could be attributed to the titanium surface features that seem to prevent bacterial colonization. This datum is similar to those reported by Pandolfi et al. [51] that estimated a prevalence of peri-implantitis from 3.2 to 9.7%.

Laser treatment was analyzed by Di Giulio et al. [52] by testing the biofilm formation of *Porphyromonas gingivalis* (in vitro) on disks made of titanium grade 4 and grade 5 with different surface topographies, and their results showed that titanium grade 4 with this laser treatment appears to be significantly less attractant for the *P. gingivalis* biofilm formation. These results were confirmed by another recent in vitro evaluation [53] comparing in vitro and in situ biofilm formation on a laser-treated titanium surface, machined, and grit-blasted. Both in vitro and in situ results demonstrated the lowest biofilm formation on laser-modified surface characterized by a few dead microbial cells.

Moreover, there is evidence that an internal conical implant-abutment connection with platform switching is an efficient factor in maintaining stable bone levels around implants in function [16]. Also, Gracis et al. [54] demonstrated that short-term results of this connection are favorable while long follow-up study are needed to evaluate long-term outcomes. The platform-switching concept proposed by Lazzara et al. [55] has been validated to reduce the peri-implant bone loss related to the microgap.

## Conclusion

Laser-treated implants with 22° conical internal hexed connection showed a maintenance of marginal bone levels over time similar (better in many cases) of what is usually reported in the literature for most commonly known implants. The low peri-implant disease incidence recorded could be attributed to the laser titanium surface features (low roughness) that seems to prevent bacterial colonization, according to several studies. The laser technique to treat dental implant allowed to create a clean and repeatable titanium surface avoiding any contamination deriving from blasting or acid procedures. According to the authors, laser-modified implants can be used successfully for various prosthetic indications. Future randomized and controlled studies are needed to confirm the results of the present multi-centrical retrospective analysis.

## Data Availability

All data and material are available from the four Italian dental centers involved in the retrospective analysis (Udine, Arezzo, Roma, Frascati).

## Abbreviations

PI: Plaque index
FMPS: Full-mouth plaque score
FMBS: Full-mouth bleeding score
FDPs: Fixed dental prostheses FDPs
BIC%: Bone to implant contact percentage
*R*_a_: Roughness value calculated on a surface (area)

## References

[1] Adler L, Buhlin K, Jansson L. Survival and complications: A 9- to 15-year retrospective follow-up of dental implant therapy. J Oral Rehabil. 2020;47:67–77. 10.1111/joor.12866 31359446

[2] Sinjari B, D'Addazio G, Traini T, Varvara G, Scarano A, Murmura G, Caputi S (2019). A 10-year retrospective comparative human study on screw-retained versus cemented dental implant abutments. J Biol Regul Homeost Agents..

[3] Salman A, Thacker S, Rubin S, Dhingra A, Ioannidou E, Schincaglia GP (2019). Immediate versus delayed loading of mandibular implant-retained overdentures: a 60-month follow-up of a randomized clinical trial. J Clin Periodontol..

[4] Gallardo YNR, da Silva-Olivio IR, Gonzaga L, Sesma N, Martin W. A systematic review of clinical outcomes on patients rehabilitated with complete-arch fixed implant-supported prostheses according to the time of loading. J Prosthodont. 2019 Aug;21. 10.1111/jopr.13104 31433096

[5] Lemos CAA, Verri FR, Gomes JML, de Souza Batista VE, Cruz RS, Oliveira HFFE, Pellizzer EP (2019). Ceramic versus metal-ceramic implant-supported prostheses: a systematic review and meta-analysis. J Prosthet Dent.

[6] Ducommun J, El Kholy K, Rahman L, Schimmel M, Chappuis V, Buser D (2019). Analysis of trends in implant therapy at a surgical specialty clinic: patient pool, indications, surgical procedures, and rate of early failures-a 15-year retrospective analysis. Clin Oral Implants Res..

[7] Dreyer H, Grischke J, Tiede C, Eberhard J, Schweitzer A, Toikkanen SE, Glöckner S, Krause G, Stiesch M (2018). Epidemiology and risk factors of peri-implantitis: a systematic review. J Periodontal Res..

[8] Bevilacqua L, Milan A, Del Lupo V, Maglione M, Dolzani L (2018). Biofilms developed on dental implant titanium surfaces with different roughness: comparison between in vitro and in vivo studies. Curr Microbiol..

[9] Berglundh T, Persson L, Klinge B (2002). A systematic review of the incidence of biological and technical complications in implant dentistry reported in prospective longitudinal studies of at least 5 years. J Clin Periodontol.

[10] Doornewaard R, Christiaens V, De Bruyn H, Jacobsson M, Cosyn J, Vervaeke S, Jacquet W (2017). Long-term effect of surface roughness and patients’ factors on crestal bone loss at dental implants. A systematic review and meta-analysis. Clin Implant Dent Relat Res..

[11] De Bruyn H, Vandeweghe S, Ruyffelaert C, Cosyn J, Sennerby L (2013). Radiographic evaluation of modern oral implants with emphasis on crestal bone level and relevance to peri-implant health. Periodontol 2000.

[12] Albrektsson T, Dahlin C, Jemt T, Sennerby L, Turri A, Wennerberg A (2014). Is marginal bone loss around oral implants the result of a provoked foreign body reaction?. Clin Implant Dent Relat Res..

[13] Tatarakis N, Bashutski J, Wang HL, Oh TJ (2012). Early implant bone loss: preventable or inevitable?. Implant Dent..

[14] De Bruyn H, Christiaens V, Doornewaard R, Jacobsson M, Cosyn J, Jacquet W, Vervaeke S (2017). Implant surface roughness and patient factors on long-term peri-implant bone loss. Periodontol 2000.

[15] Park YS, Lee SP, Han CH, Kwon JH, Jung YC (2010). The microtomographic evaluation of marginal bone resorption of immediately loaded scalloped design implant with various microthread configurations in canine mandible: pilot study. J Oral Implantol..

[16] Caricasulo R, Malchiodi L, Ghensi P, Fantozzi G, Cucchi A (2018). The influence of implant-abutment connection to peri-implant bone loss: a systematic review and meta-analysis. Clin Implant Dent Relat Res..

[17] Javed F, Romanos GE (2019). Chronic hyperglycemia as a risk factor in implant therapy. Periodontol 2000.

[18] Javed F, Rahman I, Romanos GE (2019). Tobacco-product usage as a risk factor for dental implants. Periodontol 2000.

[19] Lee D, Sohn B, Kim KH, Kim S, Koo KT, Kim TI, Seol YJ, Lee YM, Rhyu IC, Ku Y (2016). Effects of untreated periodontitis on osseointegration of dental implants in a Beagle Dog Model. J Periodontol..

[20] Trisi P, Berardini M, Falco A, Podaliri VM (2016). New osseodensification implant site preparation method to increase bone density in low-density bone: in vivo evaluation in sheep. Implant Dent..

[21] Nicolielo LFP, Van Dessel J, Jacobs R, Quirino Silveira Soares M, Collaert B. Relationship between trabecular bone architecture and early dental implant failure in the posterior region of the mandible. Clin Oral Implants Res. 2020;31:153–61. 10.1111/clr.13551 31654422

[22] Trisi P, Berardini M, Falco A, Podaliri VM (2015). Effect of implant thread geometry on secondary stability, bone density, and bone-to-implant contact: a biomechanical and histological analysis. Implant Dent..

[23] Yang J, Zhou Y, Wei F, Xiao Y (2016). Blood clot formed on rough titanium surface induces early cell recruitment. Clin Oral Implants Res..

[24] Wennerberg A (2009). Albrektsson T Effects of titanium surface topography on bone integration: a systematic review. Clin Oral Implants Res..

[25] Trisi P, Berardini M, Falco A, Sandrini E, Vulpiani MP (2017). A new highly hydrophilic electrochemical implant titanium surface: a histological and biomechanical in vivo study. Implant Dent..

[26] Smeets R, Stadlinger B, Schwarz F, Beck-Broichsitter B, Jung O, Precht C, Kloss F, Gröbe A, Heiland M, Ebker T (2016). Impact of dental implant surface modifications on osseointegration. Biomed Res Int..

[27] Berardi D, Colagiovanni M, Scoccia A, Raffaelli L, Manicone PF, Perfetti G (2008). Evaluation of a new laser surface implant: scanning electron microscopy/energy dispersive X-ray and X-ray photoelectron spectroscopy analyses. J Biol Regul Homeost Agents..

[28] Drago L, Bortolin M, De Vecchi E, Agrappi S, Weinstein RL, Mattina R, Francetti L (2016). Antibiofilm activity of sandblasted and laser-modified titanium against microorganisms isolated from peri-implantitis lesions. J Chemother..

[29] Cei S, Karapetsa D, Aleo E, Graziani F (2015). Protein adsorption on a laser-modified titanium implant surface. Implant Dentistry.

[30] Misch CE, Perel ML, Wang HL, Sammartino G, Galindo-Moreno P, Trisi P, Steigmann M, Rebaudi A, Palti A, Pikos MA, Schwartz-Arad D, Choukroun J, Gutierrez-Perez JL, Marenzi G, Valavanis DK (2008). Implant success, survival, and failure: the International Congress of Oral Implantologists (ICOI) Pisa Consensus Conference. Implant Dent..

[31] Zeza B, Pilloni A, Tatakis DN, Mariotti A, Di Tanna GL, Mongardini C (2017). Implant patient compliance varies by periodontal treatment history. J Periodontol.

[32] Sammons RL, Lumbikanonda N, Gross M, Cantzler P (2005). Comparison of osteoblast spreading on microstructured dental implant surfaces and cell behavior in an explant model of osseointegration. A scanning electron microscopic study. Clin Oral Implants Res..

[33] Zhao G, Raines AL, Wieland M, Schwartz Z, Boyan BD (2007). Requirement for both micron- and submicron scale structure for synergistic responses of osteoblasts to substrate surface energy and topography. Biomaterials..

[34] Adell R, Lekholm U, Rockler B, Brånemark PI (1981). A 15-year study of osseointegrated implants in the treatment of the edentulous jaw. Int J Oral Surg..

[35] Francetti L, Romeo D, Corbella S, Taschieri S, Del Fabbro M (2012). Bone level changes around axial and tilted implants in full-arch fixed immediate restorations. Interim results of a prospective study. Clin Implant Dent Relat Res..

[36] Felice P, Barausse C, Blasone R, Favaretto G, Stacchi C, Calvo M, Marin C, Buti J, Esposito M (2014). A comparison of two dental implant systems in partially edentulous patients: 1-year post-loading results from a pragmatic multicentre randomised controlled trial. Eur J Oral Implantol.

[37] Zuffetti F, Testarelli L, Bertani P, Vassilopoulos S, Testori T, Guarnieri R. A retrospective multicenter study on short implants with a laser-microgrooved collar (≤7.5 mm) in posterior edentulous areas: radiographic and clinical results up to 3 to 5 years. J Oral Maxillofac Surg. 2020;78:217–27. 10.1016/j.joms.2019.08.007 31518549

[38] Acharya A, Leung MCT, Ng KT, Fan MHM, Fokas G, Mattheos N (2019). Peri-implant marginal bone loss rate pre- and post-loading: an exploratory analysis of associated factors. Clin Oral Implants Res..

[39] Laurell L, Lundgren D (2011). Marginal bone level changes at dental implants after 5 years in function: a meta-analysis. Clin Implant Dent Relat Res.

[40] Vervaeke S, Collaert B, Cosyn J, Deschepper E, De Bruyn H (2015). A multifactorial analysis to identify predictors of implant failure and peri-implant bone loss. Clin Implant Dent Relat Res.

[41] Del Fabbro M, Ceresoli V (2014). The fate of marginal bone around axial vs. tilted implants: a systematic review. Eur J Oral Implantol.

[42] Sinjari B, Traini T, Caputi S, Mortellaro C, Scarano A (2018). Evaluation of fibrin clot attachment on titanium laser-conditioned surface using scanning electron microscopy. J Craniofac Surg..

[43] Quabius ES, Ossenkop L, Harder S, Kern M (2012). Dental implants stimulate expression of Interleukin-8 and its receptor in human blood--an in vitro approach. J Biomed Mater Res B Appl Biomater.

[44] Salvi GE, Aglietta M, Eick S, Sculean A, Lang NP, Ramseier CA (2012). Reversibility of experimental peri-implant mucositis compared with experimental gingivitis in humans. Clin Oral Implant Res.

[45] Aguirre-Zorzano LA, Vallejo-Aisa FJ, Estefanía-Fresco R (2013). Supportive periodontal therapy and periodontal biotype as prognostic factors in implants placed in patients with a history of periodontitis. Med Oral Patol Oral Cir Bucal.

[46] Albrektsson T, Zarb GA (1993). Current interpretations of the osseointegrated response: clinical significance. Int J Prosthodont.

[47] Trisi P, Berardini M, Colagiovanni M, Berardi D, Perfetti G (2016). Laser-treated titanium implants: an in vivo histomorphometric and biomechanical analysis. Implant Dent..

[48] De Tullio I, Berardini M, Di Iorio D, Perfetti F, Perfetti G. Comparative evaluation among laser-treated, machined, and sandblasted/acid-etched implant surfaces: an in vivo histologic analysis on sheep. Int J Implant Dent. 2020;6(1):7. 10.1186/s40729-019-0204-4 PMC7028891 32072319

[49] Krebs M, Kesar N, Begić A, von Krockow N, Nentwig GH, Weigl P (2019). Incidence and prevalence of peri-implantitis and peri-implant mucositis 17 to 23 (18.9) years postimplant placement. Clin Implant Dent Relat Res..

[50] Pjetursson BE, Thoma D, Jung R, Zwahlen M, Zembic A (2012). A systematic review of the survival and complication rates of implant-supported fixed dental prostheses (FDPs) after a mean observation period of at least 5 years. Clin Oral Implants Res..

[51] Pandolfi A, Rinaldo F, Pasqualotto D, Sorrentino F, La Torre G, Guerra F. A retrospective cohort study on peri-implant complications in implants up to 10 years of functional loading in periodontally compromised patients. J Periodontol. 2019;10.1002/JPER.18-0715. [Published online ahead of print, 2019 Dec 20]. 10.1002/JPER.18-0715 31860130

[52] Di Giulio M, Traini T, Sinjari B, Nostro A, Caputi S, Cellini L (2016). Porphyromonas gingivalis biofilm formation in different titanium surfaces, an in vitro study. Clin Oral Implants Res..

[53] Ionescu AC, Brambilla E, Azzola F, Ottobelli M, Pellegrini G, Francetti LA (2018). Laser microtextured titanium implant surfaces reduce in vitro and in situ oral biofilm formation. PLoS One..

[54] Gracis S, Michalakis K, Vigolo P, Vult von Steyern P, Zwahlen M, Sailer I (2012). Internal vs. external connections for abutments/reconstructions: a systematic review. Clin Oral Implants Res.

[55] Lazzara RJ, Porter SS (2006). Platform switching: a new concept in implant dentistry for controlling postrestorative crestal bone levels. Int J Periodontics Restorative Dent..

